# Estimating Receptive Fields from Responses to Natural Stimuli with Asymmetric Intensity Distributions

**DOI:** 10.1371/journal.pone.0003060

**Published:** 2008-08-26

**Authors:** Nicholas A. Lesica, Toshiyuki Ishii, Garrett B. Stanley, Toshihiko Hosoya

**Affiliations:** 1 RIKEN Brain Science Institute, Wako-shi, Saitama, Japan; 2 School of Engineering & Applied Sciences, Harvard University, Cambridge, Massachusetts, United States of America; 3 Toho University, Funabashi-shi, Chiba, Japan; 4 Coulter Department of Biomedical Engineering, Georgia Institute of Technology & Emory University, Atlanta, Georgia, United States of America; James Cook University, Australia

## Abstract

The reasons for using natural stimuli to study sensory function are quickly mounting, as recent studies have revealed important differences in neural responses to natural and artificial stimuli. However, natural stimuli typically contain strong correlations and are spherically asymmetric (i.e. stimulus intensities are not symmetrically distributed around the mean), and these statistical complexities can bias receptive field (RF) estimates when standard techniques such as spike-triggered averaging or reverse correlation are used. While a number of approaches have been developed to explicitly correct the bias due to stimulus correlations, there is no complementary technique to correct the bias due to stimulus asymmetries. Here, we develop a method for RF estimation that corrects reverse correlation RF estimates for the spherical asymmetries present in natural stimuli. Using simulated neural responses, we demonstrate how stimulus asymmetries can bias reverse-correlation RF estimates (even for uncorrelated stimuli) and illustrate how this bias can be removed by explicit correction. We demonstrate the utility of the asymmetry correction method under experimental conditions by estimating RFs from the responses of retinal ganglion cells to natural stimuli and using these RFs to predict responses to novel stimuli.

## Introduction

Traditionally, the response properties of sensory neurons have been studied using simple stimuli such as bars and sinusoidal gratings for vision, and clicks or pure tones for audition. More recently, the range of stimuli used to probe sensory function has been expanded to include more complex stimuli such as Gaussian white noise. While studies of responses to such artificial stimuli have provided the foundation for our understanding of sensory function, recent studies suggest that there may be fundamental differences between the neural responses to artificial stimuli and natural stimuli. For example, numerous studies have shown that natural stimuli are coded more efficiently than artificial stimuli in both the visual [1]–[4] and auditory [5]–[8] systems. Furthermore, there is evidence that models of sensory processing derived from responses to artificial stimuli are not sufficient to predict neural responses to natural stimuli [9]–[11]. These results suggest that if we hope to understand sensory function under natural conditions, we must study neural responses to natural stimuli directly.

Natural visual and auditory stimuli have complex statistical properties. For example, natural stimuli typically contain strong correlations, evidenced by power that decreases with increasing spatiotemporal or spectrotemporal modulation frequency as 1/f^α^, with α typically between 1 and 3 [12]–[15]. Natural stimuli are also spherically asymmetric, meaning that the probability distribution of stimulus intensities is not symmetric about the mean intensity (in contrast to, for example, Gaussian white noise) [13]–[17]. Unfortunately, these same complex statistical properties that differentiate natural stimuli from artificial stimuli also complicate the use of neural responses to natural stimuli in fitting models of sensory processing. With the most popular methods for characterizing sensory processing, reverse-correlation and spike-triggered averaging, an estimate of the linear filter or receptive field (RF) that provides the minimum mean squared error prediction of the neural response is computed as a weighted average of all stimuli, with each stimulus scaled by the magnitude of the response that it evoked. While these methods have proven extremely useful for characterizing the basic function of sensory systems (for a recent review, see [18]), they require that the stimulus is drawn from a spherically symmetric distribution in order to produce an unbiased RF estimate [18]–[22]. While this constraint may be satisfied by artificial stimuli such as Gaussian white noise, it is violated by the correlations and asymmetries typically found in natural stimuli, and, thus, under certain conditions, reverse correlation RF estimates computed from responses to natural stimuli can be biased.

A number of least-squares techniques in which the second-order stimulus correlations are essentially ‘divided out’ have been developed and used to estimate RFs from the responses of visual and auditory neurons to natural stimuli (for reviews, see [21], [23]–[25]). In addition to correcting for the second-order correlations in the stimulus, these approaches also correct for asymmetries in the stimulus that are due to these correlations, but other asymmetries that remain can bias the RF estimate. These effects were demonstrated in a recent simulation study that showed that even for a system consisting only of a cascade of a linear RF and a simple threshold nonlinearity, the interaction between higher-order correlations in the stimulus and the nonlinearity can lead to a biased RF estimate [26].

From an intuitive perspective, the bias in reverse correlation RF estimates caused by spherical asymmetries in the stimulus is similar to the error that would result from non-uniform stimulus sampling in a simple experiment. For example, in an attempt to characterize the ocular dominance of a neuron in the visual cortex based on the total number of spikes elicited by stimulation of each eye, it is clear that each eye must be stimulated the same number of times. If the number of stimuli presented to each eye is different, then the results must be explicitly corrected by dividing the total number of spikes elicited by stimulation of each eye by the number of times the eye was stimulated. A similar approach can be used to correct the bias in reverse correlation RF estimates spherical asymmetries in the stimulus.

Here, we develop a method for RF estimation from responses to natural stimuli that corrects for the biases introduced by spherical asymmetries by explicitly weighting the contribution of each stimulus to the RF estimate not only by the response it evokes, but also by its probability of occurrence relative to other stimuli with the same magnitude (vector norm). Through a series of simple examples using simulated neural responses, we illustrate how stimulus asymmetries can bias reverse correlation RF estimates (even for uncorrelated stimuli) and demonstrate how explicit correction for spherical asymmetries can remove this bias. We also demonstrate the application of the asymmetry correction method to experimental data by estimating the temporal RFs of retinal ganglion cells from responses to correlated, spherically asymmetric natural luminance sequences and using the RFs to predict responses to novel stimuli.

## Analysis

In this section, we establish a linear-nonlinear (LN) response model and describe the conditions under which the reverse correlation technique provides an accurate RF estimate within this context. We show how stimulus correlations and asymmetries can bias reverse correlation RF estimates and detail a method for removing these biases.

### A linear-nonlinear model for neural responses

We assume an LN model where the neural response is given by *r_i_* = *f* (*s_i_^T^ g*), where *r_i_* is the instantaneous firing rate of the neuron at time *i*, *f* (·) is a static nonlinear function,
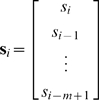
is the vector of the *m* most recent stimuli (we refer to *m* as the stimulus dimensionality), and
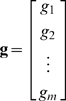
is the time-invariant temporal RF. We assume that the stimulus is wide-sense stationary (i.e., its first- and second-order statistical properties are not changing over time) and has zero mean
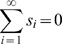



A record of *n* stimulus/response observations can be summarized as:
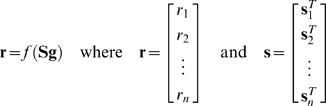
(1)are the vector of neural responses at each time and the matrix of stimulus vectors, respectively. Note that in the case where the input to the static nonlinear function *f* (·) is a vector, its output is also a vector of the same dimension.

### Reverse correlation receptive field estimation

If the system were linear (**r** = **S g**), the optimal estimate of the RF **ĝ** ^*^ (i.e. that which minimizes the mean squared error between the actual response and that predicted by the RF estimate) would be given by
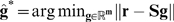
where ∥·∥ denotes vector norm (see equation 4). Many authors have shown that the solution to this equation is
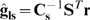
(2)where **C_S_** = **S** *^T^* **S** is the autocovariance matrix of the stimulus (see, for example, [23]). For uncorrelated stimuli with **C_S_** proportional to the identity matrix, the reverse correlation estimate of the RF
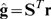
(3)will be proportional to the optimal solution **ĝ** ^*^. It has also been shown that this solution holds for the LN system described above, provided that the stimulus has zero mean and is drawn from a well sampled spherically symmetric distribution, i.e. all stimuli with the same ℓ^2^-norm
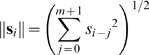
(4)must occur with the same probability
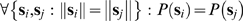
(5)and that the shape of the static NL is such that average stimulus evoking a response is non-zero (for proof, see [19]). Thus, for reverse correlation to provide an accurate RF estimate within the context of the LN model, the stimulus must be uncorrelated and spherically symmetric.

**Figure 1:**
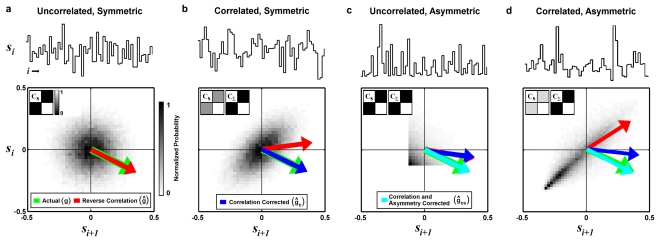
RF estimates from simulated responses to correlated and asymmetric stimuli. a) 60 sample segment of an uncorrelated and symmetric Gaussian white noise stimulus (top) along with its two-point intensity distribution (bottom) and autocovariance matrix C_S_ (bottom inset). The actual RF g = [0.3, −0.15] (*m* = 2) used to simulate LN model responses to the stimulus (green arrow) is shown along with the reverse correlation estimate of the RF ĝ computed from the simulated responses (red arrow). b) Results for a correlated Gaussian noise stimulus, presented as in a, along with the autocovariance matrix of the transformed stimulus C_Σ_ and the reverse correlation RF estimate after correction for stimulus correlations ĝ_c_ (blue arrow). c) Results for an uncorrelated stimulus drawn from an exponential distribution, presented as in b, along with the reverse correlation RF estimate after correction for stimulus correlations and asymmetries ĝ_cs_ (cyan arrow). d) Results for a correlated and asymmetric stimulus, a time-series of natural intensities taken from the database of van Hateren [16], presented as in c.

The utility of the reverse correlation approach is illustrated in the simple example shown in figure 1a. In this example, the stimulus is a Gaussian white noise sequence (representing, for example, luminance changes in a spatially uniform visual stimulus or the temporal modulations in a pure tone auditory stimulus). The Gaussian white noise stimulus is uncorrelated and spherically symmetric, as evidenced by the two-point stimulus intensity distribution and autocovariance matrix **C_S_** shown in figure 1a. We simulate responses to this stimulus using the LN model (equation 1), with **g** = [0.3, −0.15] (representing a weighted temporal summation of *m* = 2 stimulus values) and *f* (·) a perfect half-wave rectifier (*f* (x) = x for x>0 and *f* (x) = 0 otherwise). Because the stimulus is uncorrelated and spherically symmetric, the reverse correlation estimate of the RF from the simulated responses **ĝ** = **S** *^T^* **r** (red arrow) matches the actual RF (green arrow).

### The effects of stimulus correlations

If the stimulus is correlated, then the reverse correlation estimate **ĝ** will be a biased version of the actual RF **g**, with the bias determined by the autocovariance matrix **C_S_**. This is illustrated in the simple example shown in figure 1b. In this example, the stimulus is a Gaussian noise sequence with strong correlations, evidenced by the skewed two-point intensity distribution and the non-zero off-diagonal elements of the autocovariance matrix **C_S_**. Because of these correlations, the reverse correlation estimate of the RF **ĝ** (red arrow) computed from responses simulated with the LN model is biased toward the elongated dimension in the stimulus distribution and does not match the actual RF **g** (green arrow).

Fortunately, the reverse correlation estimate **ĝ** can be modified to correct the bias due to the stimulus correlations. For example, the reverse correlation estimate can be multiplied by the inverse of the autocovariance matrix to produce the least-squares RF estimate **ĝ** **_ls_** as described above. Indeed, this correction has been used to estimate RFs from responses to natural stimuli in many brain areas [9], [11], [27]–[31]. An alternative approach is to transform the stimulus to remove correlations before computing the reverse correlation estimate [3], [4]. For the LN model neuron described above we can transform the stimulus as follows:
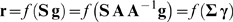
(6)where **Σ** = **S A** and **γ** = **A** ^−1^ **g**. We can compute the reverse correlation estimate of the transformed RF **γ** from the transformed stimulus **Σ** and the response **r**, **γ̂** = **Σ** *^T^* **r**, and obtain an estimate of the actual RF **g** by inverting the transformation on the RF, **ĝ** **_c_** = **A** **γ̂**. The matrix **A** is chosen such that the transformed stimulus is uncorrelated (i.e. its autocovariance matrix is proportional to the identity matrix). The autocovariance matrix of the transformed stimulus **C_Σ_** can be written as:
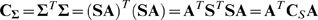
Thus, we want to choose **A** such that **A** *^T^* **C_S_** **A** = **I**. **C_S_** can be decomposed as **C_S_** = **V DV** ^−1^ where **V** is an orthogonal matrix of the eigenvectors of **C_S_** (i.e. the principal components of **S**) with **V** *^T^* = **V** ^−1^ and **D** is a diagonal matrix with the corresponding eigenvalues [λ_1_^2^, λ_2_^2^, …, λ*_m_* ^2^] on the diagonal (or, alternatively, a similar representation can be obtained through singular value decomposition as described in [32]). If we choose **A** = **V** (**D** ^−1/2^)*^T^* , then **C_Σ_** = **A** *^T^* **C_S_** **A** = **D** ^−1/2^ **V** *^T^* (**V** **DV** ^−1^) **V** (**D** ^−1/2^)*^T^* = **I** and the transformed reverse correlation estimate
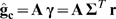
(7)will not be biased by the stimulus correlations.

To demonstrate the utility of this correction, we return to the simple example of estimating a known RF from simulated responses to a correlated Gaussian noise stimulus shown in figure 1b. As described above, because of the correlations in the stimulus, the reverse correlation estimate **ĝ** is biased. However, if we apply the transformation described in equation 6 to correct for the stimulus correlations, then the autocovariance matrix of the transformed stimulus **C_Σ_** has zero off-diagonal elements and the resulting RF estimate **ĝ** **_c_**(blue arrow) now matches the actual RF **g**.

Note that the transformed reverse correlation estimate **ĝ** **_c_** = **A** **Σ** *^T^* **r** can also be written as **ĝ** **_c_** = **A** **A** *^T^* **S** *^T^* **r** = **V**(**D** ^−1/2^)*^T^* **D** ^−1/2^ **V** *^T^* **S** *^T^* **r**. In this form, it is clear that in computing the transformed reverse correlation estimate, each principal component of **S** is multiplied by a factor related to the inverse of the corresponding eigenvalue. Thus, those principal components with smallest eigenvalues will have the largest effect on the estimate. For high dimensional natural stimuli with strong correlations, the difference between the largest and smallest eigenvalues can be several orders of magnitude (i.e. the condition number of the stimulus autocovariance matrix can be extremely large), and the effect of the principal component with largest eigenvalue on the RF estimate can be dwarfed by that of the principal component with the smallest eigenvalue. In this case, the RF estimate will be largely determined by those principal components along which the stimulus has the smallest variance and, under experimental conditions where only a limited number of noisy responses are observed, a large difference in eigenvalues can result in an RF estimate that is dominated by noise.

A number of approaches have been proposed to address this problem. For example, Theunissen and colleagues [9], [23] have computed the transformed RF estimate using only those principal components with eigenvalues larger than some threshold value (i.e. if an eigenvalue is less than the threshold value, then the element of **D** ^−1/2^ corresponding to that eigenvalue is set to zero). We adopted a variant of this approach in which only those principal components required to explain a certain fraction 0<ε≤1 of the variance in the stimulus were retained. In addition to reducing the noise in the transformed reverse correlation RF estimate, eliminating some fraction of the principal components allows the stimulus to be represented in a lower dimensional space m*≤*m*, which simplifies the estimation of the stimulus probability distribution *P*(**s**) as described below.

### The effects of stimulus asymmetries

The transformation described above corrects the bias in reverse correlation RF estimates due to the second-order correlations in the stimulus, as well as the bias due to any asymmetries in the stimulus intensity distribution that result from those correlations. However, complex stimuli can contain additional asymmetries and these asymmetries can also bias RF estimates. This is illustrated in the simple example shown in figure 1c. In this example, each value of the stimulus is drawn from an uncorrelated exponential distribution, and there are clear asymmetries in the two-point intensity distribution. Because of these asymmetries, the reverse correlation estimate of the RF **ĝ** (red arrow) computed from simulated response of the LN model is biased and does not match the actual RF (green arrow). Additionally, because the stimulus autocovariance matrix **C_S_** is already proportional to the identity matrix, applying the transformation described in equation 6 to correct for the stimulus correlations has no effect, and the transformed reverse correlation RF estimate **ĝ** **_c_**(blue arrow) is also biased.

Fortunately, the bias in the RF estimate due to stimulus asymmetries can also be corrected. Conceptually, the correction necessary to remove the bias due to stimulus asymmetries is analogous to the transformation used to correct the bias due to stimulus correlations. In removing the bias due to stimulus correlations, each principal component is weighted by the inverse of the amount of stimulus variance that it explains, such that the effective contribution of every principal component to the transformed stimulus is the same. Similarly, the bias due to stimulus asymmetries can be removed by weighting each stimulus by its probability of occurrence relative to those of other stimuli with the same vector norm, such that the effective probability distribution of the stimulus is spherically symmetric. If we want to correct the biases due to stimulus correlations and asymmetries simultaneously, then we can estimate the RF as:
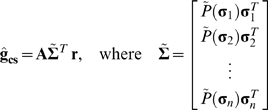
(8)where *P̃*(**σ** *_i_*) = *P̅*(∥**σ** *_i_*∥) *P*(**σ** *_i_*)^−1^ is the asymmetry correction for a particular (transformed) stimulus and *P̅*(∥**σ** *_i_*∥) is the mean probability of occurrence of all stimuli with same vector norm as stimulus **σ** *_i_*.

The asymmetry correction described in equation 8 requires estimation of the overall probability distribution of the stimulus *P*(**σ**) and norm-specific probabilities *P̅*(∥**σ** *_i_*∥). In practice, we estimate *P*(**σ**) by grouping stimuli into evenly spaced bins that span the range of stimulus values. *P*(**σ**) is estimated not from the original stimulus, but from the coefficients that define each stimulus within the space defined by the principal components in **V**, thus reducing the dimensionality of *P*(**σ**) from *m* to m* (note that for natural stimuli, which typically contain strong correlations, the value of m* that results in the best RF estimates is often much less than *m*). The norm-specific probabilities *P̅*(∥**σ** *_i_*∥) are estimated by dividing the range of norms into evenly spaced bins and taking the mean of the probabilities of all stimuli whose norms fall within each bin. In general, we found that the effect of the correction for stimulus asymmetries was robust to changes in the number of bins used to estimate *P*(**σ**) and *P̅*(∥**σ** *_i_*∥), even for relatively high-dimensional problems. Thus, for all of the simulated and experimental examples below, the probability distribution of the stimulus *P*(**σ**) was computed after dividing the range of stimulus values into 250 evenly spaced bins and the norm-specific probabilities *P̅*(∥**σ** *_i_*∥) were computed after dividing the range of norms into 250 evenly spaced bins.

To demonstrate the utility of the asymmetry correction described in equation 8, we return to the simple example of estimating a known RF from simulated responses to a stimulus drawn from an uncorrelated exponential distribution shown in figure 1c. As described above, because of the asymmetries in the stimulus, both the reverse correlation estimate **ĝ** and the transformed reverse correlation estimate **ĝ** **_c_** are biased. However, if we apply the asymmetry correction described in equation 8, then the resulting RF estimate **ĝ** **_cs_** = **A** **Σ̃** *^T^* **r** (cyan arrow) now matches the actual RF (green arrow).

### Temporal receptive field estimation from simulated responses to natural stimuli

A final simple example shown in figure 1d demonstrates the utility of the asymmetry correction for a stimulus that is both correlated and asymmetric. In this example, the stimulus is a time-series of natural intensities taken from the database of van Hateren [16]. This stimulus contains strong correlations and asymmetries, as illustrated in two-point intensity distribution and stimulus autocovariance matrix **C_S_** shown in figure 1d. Because of these correlations and asymmetries, the reverse correlation estimate of the RF **ĝ** (red arrow) computed from simulated responses of the LN model is biased and does not match the actual RF **g** (green arrow). Transformation of the stimulus to remove the bias due to correlations improves the estimate **ĝ** **_c_**(blue arrow), but only after correction for for both stimulus correlations and asymmetries does the estimate **ĝ** **_cs_** (cyan arrow) match the actual RF.

The simple examples in figure 1 demonstrate the ability of the asymmetry correction to improve estimates of low-dimensional (*m* = 2) RFs. However, the RFs estimated from experimental responses of sensory neurons data typically have a much higher dimensionality (∼10<*m*<∼1000). Because the asymmetry correction depends on the estimation of the *m* ^*^-dimensional probability distribution of the stimulus *P*(**σ**), the efficacy of the correction may decrease as *m* increases.

To improve the efficacy of the asymmetry correction for higher-dimensional RFs, we added two additional parameters. The first parameter, θ, specifies the vector norm threshold that determines whether stimuli and their corresponding responses are included or excluded from the RF estimate (only stimuli with vector norms below θ are included in the estimate). The second parameter, φ, specifies the maximum value for the asymmetry correction *P̃*(**σ** *_i_*). After rescaling the asymmetry corrections for all stimuli such that 1≤*P̃*(**σ** *_i_*)<∞, any *P̃*(**σ** *_i_*)>φ are set equal to φ. For natural stimuli, the probability of a stimulus *P*(**σ**) tends to decrease with increasing norm and excluding those low probability stimuli with large norms or limiting the value of their asymmetry correction *P̃*(**σ** *_i_*) can improve the efficacy of the overall asymmetry correction.

Including the two new parameters θ and φ, the asymmetry correction can be written as
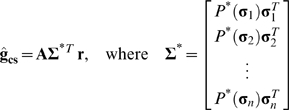
(9)where *P* ^*^(**σ** *_i_*) = min (φ, *Ν_i_ P̃*(**σ** *_i_*)) and *Ν_i_* = 1 if ∥**σ** *_i_*∥≤θ and 0 otherwise. In all of the examples below, we test the efficacy of the asymmetry correction for a range of values for θ and φ, including θ = ∞ and φ = 1, the values for which the asymmetry correction has no effect and the RF estimate **ĝ** **_cs_** is equivalent to the RF estimate that is corrected for correlations only **ĝ** **_c_**.

**Figure 2:**
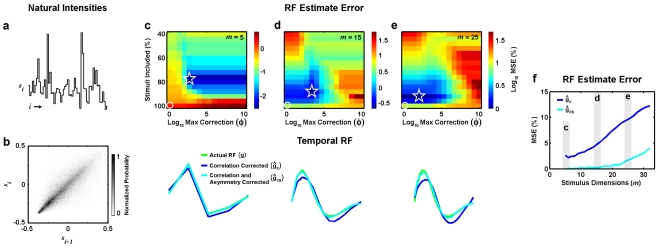
Temporal RF estimates from simulated responses to natural stimuli. a) 60 sample segment of a time-series of natural intensities taken from the database of van Hateren [16]. b) Two-point distribution of intensities in the natural stimulus. c) Color image showing the (log_10_) mean squared error between the actual RF used to simulate responses of the LN model to the natural stimulus and RF estimates corrected for stimulus correlations and asymmetries ĝ_cs_ computed from the simulated responses with different values of θ, defined in terms of the % of stimuli that were included in the RF estimate, and φ, the maximum value for the asymmetry correction. The minimum error is denoted by the star, and the error corresponding to correction for correlations only is denoted by the circle. The actual RF (*m* = 5) used to simulate the LN model responses to the natural stimulus is shown (green), along with the RF estimated from simulated responses after correction for stimulus correlations only ĝ_c_ (blue) and correction for stimulus correlations and asymmetries ĝ_cs_ with optimal values of θ and φ (cyan). d,e) Results for *m* = 15 and *m* = 25, presented as in c. f) Mean squared error between actual RFs and RF estimates ĝ_c_ (blue) and ĝ_cs_ with optimal values of θ and φ (cyan) for different values of *m*.

As a first test of the efficacy of the asymmetry correction for higher-dimensional RFs, we used the LN model to simulate responses to the time-series of natural intensities (shown again in figure 2a, along with its two-point intensity distribution in figure 2b), but for these simulations the RF was chosen to have a biphasic shape that is typical of temporal RFs in early sensory systems. We repeated the simulation while increasing the dimensionality of the stimulus from *m* = 5 to a more realistic value of *m* = 32 (representing, for example, temporal summation of 32 frames of a spatially uniform visual stimulus).

To explicitly examine the bias in the RF estimate due to stimulus asymmetries and the efficacy of the asymmetry correction, we compared the actual RF **g** to the RF estimated from simulated responses to the natural stimulus after correcting for correlations only **ĝ** **_c_** and after correcting for both correlations and asymmetries **ĝ** **_cs_** with optimal values for θ and φ. Note that for all of the simulations shown in figure 2, the best estimates of **ĝ** **_cs_** and **ĝ** **_c_** were obtained when all principal components of the stimulus were used (ε = 1 and *m* ^*^ = *m*) and, thus, only results obtained with these values are shown.

The results for an RF with *m* = 5 are shown in figure 2c. The color image shows the bias in **ĝ** **_cs_** (defined as the mean squared error between **g** and **ĝ** **_cs_**) for different values of θ and φ. The estimate of the RF with the lowest error (denoted by the star) was obtained when θ was set such that the stimuli with the smallest 78% of norms were included in the estimate and the maximum asymmetry correction was set to φ = 10^3^. For these optimal values of θ and φ, the RF estimate **ĝ** **_cs_** (shown in cyan) is identical to the actual RF (shown in green). The error in the estimate increases when the RF estimate is corrected for correlations only (θ = ∞ and φ = 1, denoted by the circle) and the RF estimate **ĝ** **_c_** (shown in blue) is biased. For *m* = 5, the bias in **ĝ** **_c_** due to stimulus asymmetries is small, but this bias increases as the dimensionality of the RF increases (shown for *m* = 15 in figure 2d and *m* = 25 in figure 2e) and the asymmetry correction substantially reduces this bias. A summary of the bias in **ĝ** **_c_** and the efficacy of the asymmetry correction across a range of values for *m* is shown in figure 2f. The bias in **ĝ** **_c_** (blue) increases steadily as the dimensionality of the stimulus increases, while the bias in **ĝ** **_cs_** (cyan) increases much more slowly.

### Spatial receptive field estimation from simulated responses to natural stimuli

**Figure 3:**
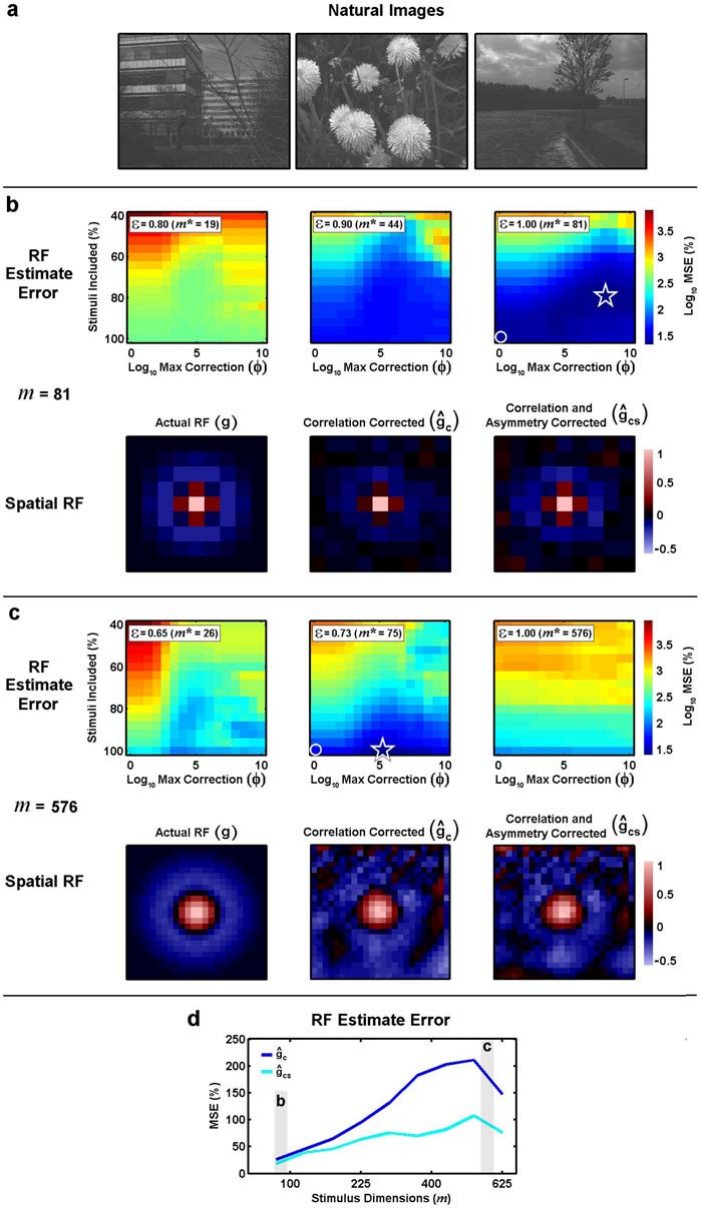
Spatial RF estimates from simulated responses to natural stimuli. a) Three example natural images taken from the database of van Hateren [33]. b) Color image showing the mean squared error between the actual RF used to simulate responses of the LN model to the natural stimulus and RF estimates corrected for stimulus correlations and asymmetries ĝ_cs_ computed from the simulated responses with different values of ε, the fraction of the variance explained by the principal components of the stimulus used in computing the RF estimate, θ, defined in terms of the % of stimuli that were included in the RF estimate, and φ, the maximum value for the asymmetry correction. The minimum error is denoted by the star, and the error corresponding to correction for correlations only is denoted by the circle. The actual RF (*m* = 81) used to simulate the LN model responses to the natural stimulus is shown, along with the RF estimated from simulated responses after correction for stimulus correlations only ĝ_c_ with an optimal value of ε, and correction for stimulus correlations and asymmetries ĝ_cs_ with optimal values of ε, θ, and φ. c) Results for *m* = 576, presented as in b. d) Mean squared error between actual RFs and RF estimates ĝ_c_ (blue) and ĝ_cs_ with optimal values of θ and φ (cyan) for different values of *m*.

When next tested the efficacy of the asymmetry correction for even higher-dimensional RFs. We used the LN model to simulate responses to a series of natural images taken from the database of van Hateren [33]. For these simulations, the RF *g* was defined by *m* points in space with a center-surround structure that is typical of spatial RFs in the early visual pathway and the stimulus **s** *_i_* was defined as
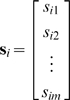
where *s_ij_* is the intensity of pixel *j* in image *i*. Several example images are shown in figure 3a. We again repeated the simulation while increasing the dimensionality of the RF from *m* = 81 to *m* = 625, and the natural images were resized to achieve the desired dimensionality.

The results for an RF with *m* = 81 are shown in figure 3b. In this example, the estimate of the RF with the lowest error (denoted by the star) was obtained when all principal components of the stimulus were used (ε = 1 and *m* ^*^ = 81) and θ was set such that the stimuli with the smallest 80% of norms were included in the estimate and the maximum asymmetry correction was set to φ = 10^8^. The error in the estimate increases when the RF estimate is corrected for correlations only (θ = ∞ and φ = 1, denoted by the circle). The RF estimate **ĝ** **_c_** that is corrected for correlations only has a weaker surround than the actual RF **g**, while the RF estimate corrected for both correlations and asymmetries **ĝ** **_cs_** and the actual RF are similar.

Figure 3c shows the results for a high-dimensional RF with *m* = 576. In this example, the estimate of the RF with the lowest error was obtained when only the principal components necessary to explain 73% of the variance in the stimulus were used (ε = 0.73 and *m* ^*^ = 75; in this example, these values were optimal for both **ĝ** **_cs_** and **ĝ** **_c_**), with all stimuli included in the estimate and the maximum asymmetry correction set to φ = 10^5^. When only correlations are corrected, the RF estimate **ĝ** **_c_** has a strong bias evidenced by the large negative values at the bottom of the RF and correcting for both correlations and asymmetries in **ĝ** **_cs_** reduces this bias. A summary of the bias in **ĝ** **_c_** and the efficacy of the asymmetry correction across a range of values *m* is shown in figure 3d. As with the temporal RF examples shown in figure 2, the bias in the spatial RF estimate after correcting for correlations only **ĝ** **_c_** (blue) increases steadily as the dimensionality of the stimulus increases, while the bias in the estimate after correcting for both correlations and asymmetries **ĝ** **_cs_** (cyan) increases much more slowly.

### Temporal receptive field estimation from experimental responses to natural stimuli

**Figure 4:**
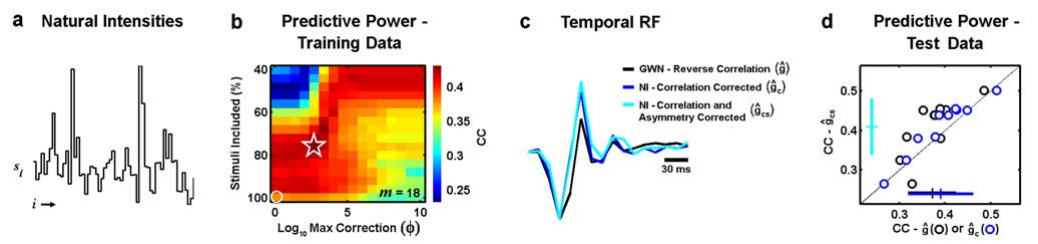
Temporal RF estimates from experimental responses to natural stimuli. a) 60 sample segment of a time-series of natural intensities taken from the database of van Hateren [16]. b) Color image showing the correlation coefficient (CC) between the experimental responses of a retinal ganglion cell to the ‘training’ segment of the natural stimulus and the responses predicted by RF estimates (*m* = 18) corrected for stimulus correlations and asymmetries ĝ_cs_ computed from the experimental responses with different values of θ, defined in terms of the % of stimuli that were included in the RF estimate, and φ, the maximum value for the asymmetry correction. The maximum CC is denoted by the star, and the CC corresponding to correction for correlations only is denoted by the circle. c) The RF estimated from experimental responses to the natural stimuli after correction for stimulus correlations only ĝ_c_ is shown (blue), along with the RF estimated from experimental responses to the natural stimuli after correction for stimulus correlations and asymmetries ĝ_cs_ with optimal values of θ and φ (cyan) and the RF estimated from experimental responses of the same cell to white noise stimuli using reverse correlation ĝ (black). d) Correlation coefficient between predicted and actual firing for responses to the ‘testing’ segment of the natural stimulus for RF estimates ĝ_c_, ĝ_cs_ with optimal values of θ and φ, and ĝ for a sample of 10 retinal ganglion cells. Crosses indicate sample mean and standard deviation.

The above results demonstrate that correcting for stimulus asymmetries can reduce the bias in an RF estimate computed from simulated responses to a natural stimulus, even for relatively high-dimensional RFs. However, under experimental conditions, the data available for RF estimation can be limited to a relatively small number of noisy observations of the neural response. To determine whether explicit correction for stimulus asymmetries is sufficient to provide accurate RF estimates under such conditions, we recorded retinal responses to the same natural time-series used in the simulated examples described in figure 2 (shown again in figure 4a). The spatially uniform stimulus was projected onto an isolated salamander retina and action potentials from ganglion cells were recorded extracellularly. The methods for these experiments have been described in detail previously [34].

The experimental responses were used to estimate RFs with correction for stimulus correlations and asymmetries as described above. Because this is an experimental situation, we do not have access to the actual RF with which to compare our estimate. Instead, we evaluate the quality of the RF estimates by measuring their ability to predict experimental responses to novel natural stimuli. First, the stimulus/response data for each neuron are divided into ‘training’ and ‘testing’ segments to avoid contamination of the evaluation from ‘overfitting’ the noise in the response. Next, the training data are used to estimate the RF and determine the optimal value of ε for **ĝ** **_c_**, and the optimal values of ε, θ, and φ for **ĝ** **_cs_**. The optimal parameter values are chosen by using the RF estimate in the LN model to simulate responses to the training stimulus (with *f* (·) also estimated from the training data as in [20]) and maximizing the predictive power measured as the correlation coefficient between the simulated and actual responses. Finally, the resulting RF estimates are used to simulate responses to the testing stimulus and the predictive power between the simulated and actual responses is measured. To provide an additional benchmark for comparison, we also recorded responses of the same cells to a spatially uniform Gaussian white noise stimulus and computed the reverse correlation RF estimate **ĝ** from these responses.

The results for an example OFF-center cell are shown in figures 4b and c with *m* = 18. For this cell, the estimate of the RF that had the highest predictive power for the training data was obtained when all principal components of the stimulus were used (ε = 1 and *m* ^*^ = 18), with θ set such that the stimuli with the smallest 77% of norms were included in the estimate and the maximum asymmetry correction set to φ = 10^3^. When only correlations are corrected, the predictive power of the RF estimate for the training data is decreased and differences between **ĝ** **_cs_** (cyan) and **ĝ** **_c_** (blue) are evident in the later phases of the RF estimates. Additionally, both **ĝ** **_cs_** and **ĝ** **_c_** are substantially different from the RF estimated from responses to the white noise stimulus **ĝ**.

To evaluate the quality of the different RF estimates, we measured their predictive power for the testing data as described above. As shown in figure 4d for a sample of 10 cells, the predictive power of the RFs estimated from responses to the natural stimulus with correction for stimulus correlations and asymmetries **ĝ** **_cs_** is significantly larger than that of the RFs estimated from responses to the natural stimulus with correction for stimulus correlations only **ĝ** **_c_**, as well as that of the RFs estimated from responses to the white noise stimulus **ĝ** (paired t-tests, p<0.01). On average, the predictive power of **ĝ** **_cs_** was 5% larger than that of **ĝ** **_c_** and 11% larger than that of **ĝ**, with increases for individual cells as large as 13% and 28%, respectively. This suggests that the RFs with correction for stimulus correlations and asymmetries do indeed provide a more accurate description of temporal processing in retinal ganglion cells than the RFs estimated with correction for stimulus correlations only and that the explicit correction for stimulus asymmetries can be effective in the analysis of experimental responses to natural stimuli.

## Discussion

We have described a method for correcting the bias in reverse correlation RF estimates that arises from the asymmetries typical of natural stimuli. Using simulated neural responses, we have illustrated how stimulus asymmetries can bias reverse correlation RF estimates (even for uncorrelated stimuli) and demonstrated how explicit correction for spherical asymmetries can remove this bias. We have also shown that this method is suitable for estimating RFs under experimental conditions using retinal responses to natural stimuli. Below, we discuss the limitations of the asymmetry correction method presented here and consider other methods for RF estimation from responses to natural stimuli.

### Limitations of the asymmetry correction

The primary limitation of the asymmetry correction method, at least in theory, is that its efficacy decreases as the stimulus dimensionality increases. Because the asymmetry correction method requires that responses to stimuli with low probability are weighted heavily in the RF estimate, the stability of the correction suffers as the stimulus dimensionality (and, thus, for natural stimuli, the percentage of stimuli with low probability) is increased. For high-dimensional stimuli, this problem is mitigated somewhat by using only a subset of the principal components of the stimulus in computing the transformation that corrects the RF estimate for second-order stimulus correlations. We further addressed this problem by introducing two additional parameters, θ and φ, into the asymmetry correction that limit the set of stimuli used in the RF estimate and the maximum value of the asymmetry correction. It is important to note that if the system is indeed well described by the LN model (the assumption upon which most RF-based analyses are based), then the exclusion of any particular subset of stimuli will not bias the RF estimate. Furthermore, as long as the range of values of θ and φ that are tested include θ = ∞ and φ = 1, then the resulting RF estimate will be at worst equivalent to the RF estimate that is corrected for correlations only.

The asymmetry correction method presented here is only useful when the neural response can be accurately described by the standard LN model. However, there are many sensory neurons with nonlinear response properties for which the standard LN model is inadequate. Several recent studies have used spike-triggered covariance (STC) techniques to estimate RFs for more sophisticated LN models containing multiple linear filters with nonlinear interactions (for review, see [18], [22], [35]). These techniques have allowed for the characterization of neural responses that are incompatible with the standard LN model, such as motion sensitive cells in the fly lobula plate and complex cells in the primary visual cortex [3], [4], [36], [37]. The correlations and asymmetries in natural stimuli can also bias estimates of the STC (indeed, the symmetry requirements for unbiased STC estimates are stricter than those for RF estimates in the standard LN model [18], [21]). It is possible that the explicit asymmetry correction introduced here could be extended to provide unbiased STC estimates from responses to natural stimuli.

The standard LN model also assumes that the structure of the RF is time-invariant. While this assumption is appropriate for many sensory neurons under stationary stimulus conditions, the natural environment can be highly nonstationary and changes in the statistical properties of the stimulus can evoke adaptive changes in neural response properties (for reviews, see [38], [39]). This adaptation is reflected in significant changes in the structure of the RF and, as a result, the standard LN model with a time-invariant RF is often insufficient to describe neural responses to nonstationary stimuli. To track adaptive changes in RF structure, we have previously extended the least-squares approach for RF estimation (see equation 2) to estimate time-varying RFs [30], [31], [40]. As described above, the least-squares approach corrects RF estimates for the bias introduced by stimulus correlations, but not for the bias introduced by stimulus asymmetries. The asymmetry correction method developed here could also be extended to estimate time-varying RFs, but the stability of the correction may depend strongly on the degree of nonstationarity in the distribution of stimulus intensities.

### Other methods for receptive field estimation from responses to natural stimuli

In a number of studies in both the visual and auditory systems, RFs have been estimated from responses to natural stimuli and the bias due to second-order stimulus correlations has been corrected using a least-squares approach (equation 2, or some variant thereof) [3], [4], [9], [11], [27]–[31]. Detailed descriptions and analyses of this approach have also been published [21], [23]–[25]. In the studies cited above, the biases in the RF estimates due to stimulus asymmetries were not explicitly corrected. However, in some of these studies, the authors attempted to quantify the bias in the RF estimates introduced by stimulus asymmetries by simulating responses to natural stimuli using an LN model with a known RF and comparing the actual RF to the RF estimated from the simulated responses [3], [4], [31], [41]. Because the actual and estimated RFs were similar, the authors concluded that the bias in the RF estimates introduced by stimulus asymmetries was minimal. In contrast, a similar comparison between RF estimates corrected for correlations only and actual RFs in our results revealed large differences (see, for example, figure 2e) and another recent simulation study found similar results [26]. Taken together, these results imply that the bias in RF estimates introduced by stimulus asymmetries is dependent on the specific statistical properties of the stimulus and suggest that these effects should be investigated explicitly in each new experimental context.

Recently, several new techniques for RF estimation have been developed that use gradient descent methods to produce RF estimates that are independent of both stimulus correlations and asymmetries. One set of techniques minimizes the same cost function used in reverse correlation RF estimates, the mean squared error between the predicted and actual responses [32], [42], while another set maximizes some variant of the mutual information between the stimulus and the predicted response [21], [43], [44]. Both sets of techniques have already been used successfully to estimate high-dimensional RFs under experimental conditions. The only potential drawback to these approaches is that they require a search algorithm to determine the optimal RF and it may be difficult to avoid local optima. Another promising new approach involves maximum likelihood estimation of a parametric LN model [45]. While this approach also requires a search algorithm to find the optimal parameters, it takes advantage of the fact that, for certain forms of the LN model, the likelihood surface is convex, allowing for an efficient search. This approach has been used successfully to characterize retinal ganglion cell responses to white noise stimuli [46], but has not yet been tested with natural stimuli.

While not explicitly biased by asymmetries in the stimulus, RFs estimated using gradient descent methods will, by definition, be most successful in minimizing or maximizing the relevant cost function for predicted responses to those stimuli that occur most frequently. Thus, these estimates are still influenced by the probability distribution of the stimulus and it is possible that they could also benefit from a correction similar to the asymmetry correction described here in which the contribution of each response to the cost function is weighted by the probability of the corresponding stimulus.
